# Critical role of kinase activity of hematopoietic progenitor kinase 1 in anti-tumor immune surveillance

**DOI:** 10.1371/journal.pone.0212670

**Published:** 2019-03-26

**Authors:** Jinqi Liu, Joshua Curtin, Dan You, Stephen Hillerman, Bifang Li-Wang, Rukiye Eraslan, Jenny Xie, Jesse Swanson, Ching-Ping Ho, Simone Oppenheimer, Bethanne M. Warrack, Colleen A. McNaney, David M. Nelson, Jordan Blum, Taeg Kim, Mark Fereshteh, Michael Reily, Petia Shipkova, Anwar Murtaza, Miguel Sanjuan, John T. Hunt, Luisa Salter-Cid

**Affiliations:** Immuno-oncology Discovery, Bristol Myers Squibb, Princeton, New Jersey, United States of America; Aix-Marseille Universite, FRANCE

## Abstract

Immunotherapy has fundamentally changed the landscape of cancer treatment. Despite the encouraging results with the checkpoint modulators, response rates vary widely across tumor types, with a majority of patients exhibiting either primary resistance without a significant initial response to treatment or acquired resistance with subsequent disease progression. Hematopoietic progenitor kinase 1 (HPK1) is predominantly expressed in hematopoietic cell linages and serves as a negative regulator in T cells and dendritic cells (DC). While HPK1 gene knockout (KO) studies suggest its role in anti-tumor immune responses, the involvement of kinase activity and thereof its therapeutic potential remain unknown. To investigate the potential of pharmacological intervention using inhibitors of HPK1, we generated HPK1 kinase dead (KD) mice which carry a single loss-of—function point mutation in the kinase domain and interrogated the role of kinase activity in immune cells in the context of suppressive factors or the tumor microenvironment (TME). Our data provide novel findings that HKP1 kinase activity is critical in conferring suppressive functions of HPK1 in a wide range of immune cells including CD4+, CD8+, DC, NK to Tregs, and inactivation of kinase domain was sufficient to elicit robust anti-tumor immune responses. These data support the concept that an HPK1 small molecule kinase inhibitor could serve as a novel agent to provide additional benefit in combination with existing immunotherapies, particularly to overcome resistance to current treatment regimens.

## Introduction

Successful anti-tumor immunity relies on a functional “cancer-immunity cycle,” including antigen processing and presentation, activation of T cells, trafficking of antigen specific T effector cells and engagement of target tumor cells by the activated T effector cells [1, 2]. However, this “cancer-immunity cycle” can be interrupted by mechanisms involved in development of tolerance and immune evasion as reflected in the typical tumor microenvironment. One of the main strategies of effective cancer immune therapy is to break peripheral tolerance to allow recognition of tumor antigen as a non-self entity and to overcome immunosuppressive factors present in the tumor microenvironment. HPK1, a member of the MAP4K family, is a hematopoietic-specific protein serine-threonine kinase. With its primary expression in hematopoietic cells, a potential regulatory role of HPK1 was suggested in mediating signaling of hematopoietic lineages [3, 4]. HPK1 KO mouse studies revealed the essential role of HPK1 in negatively regulating T cell activation with involvement of the linker of activated T cells (LAT) and associated downstream signaling molecules, including adaptor protein Src homology 2 (SH2) domain containing leukocyte protein of 76 kDa (SLP-76), phospholipase Cγ1 (PLCγ1) and extracellular signal–regulated kinases (ERKs) signaling pathway. HPK1 ablation resulted in enhanced T cell activation and immune functions, as demonstrated by the increased susceptibility of HPK1 KO mice to experimental autoimmune encephalomyelitis [5]. HPK1 mRNA and protein levels were observed to be significantly decreased in CD4+ T cells of patients with systemic lupus erythematosus (SLE), contributing to the overactivation of T and B cells in SLE [6], supporting an important role of HPK1 in the maintenance of peripheral tolerance. Consistently, studies also demonstrated that mice received adoptive transfer of HPK1 KO T cells became resistant to Lewis lung carcinoma (LLC) tumor growth via mounting effective anti-tumor immune responses [7], suggesting that inhibition of HPK1 could be a viable approach for cancer immune therapy by promoting effector functions of T cells. Moreover, tumor evasion is one of the key challenges in current cancer immunotherapy [8]. Of known tumor evasive mechanisms, production of immunosuppressive factors by tumor cells is recognized as an effective way for tumor cells to disarm anti-tumor immunity. One of the hallmarks of various cancer cells is the aberrant expression of cyclooxygenase 2 (COX-2) [9–12], a key enzyme involved in the metabolism of arachidonic acid and generation of prostaglandin E2 (PGE2), an immune suppressive factor that initiates signals to block T-cell proliferation and secretion of proinflammatory cytokines [13–15]. Previous studies demonstrated that HPK1 KO augmented the growth resistance of PGE2-producing Lewis lung carcinoma. HPK1 KO T cells were demonstrated to be resistant to PGE2-mediated suppression of T-cell proliferation, IL-2 production, and apoptosis, suggesting that PGE2 utilizes HPK1, at least in part, to suppress T cell-mediated anti-tumor responses [16]. With extensive data indicating a key role of HPK1 in mediating immune tolerance and resistance to immune suppressive factor such as PGE2 using HPK1 KO mice and the potential scaffolding role of HPK1, the contribution of its kinase activity to these observations remains unknown. In this study, we generated HPK1 KD mice by introducing a point mutation (Lys46Met = K46M) on the key residue responsible for HPK1 kinase activity. Extensive characterization of these mice revealed a critical role of HPK1 kinase activity in mediating immune cell functions, anti-tumor immunity as well as resistance to immune suppressive factors including PGE2 and adenosine. Our data provide novel evidence that blockade of HPK1 kinase activity is sufficient to beneficial effect on enabling an optimal “cancer-immunity cycle”, supporting that pharmacological intervention of HPK1 kinase activity could serve as a novel immunomodulatory approach to anticancer therapy.

## Materials and methods

### Animals

Wild type and HPK1 kinase dead mice from The Jackson Laboratory (Bar Harbor, ME) were received at age 8–9 weeks and acclimated for 3–7 days prior to tumor implantation. All mice were maintained at 70–74°F and 40–60% relative humidity, with a 12:12 hour light:dark cycle. Animals were fed Teklad Global Diets 2918 (Envigo, Madison, WI) ad libitum and were housed on ALPHA-dri (Shepard Specialty Papers, Milford, NJ) bedding in ventilated caging.

All animal procedures were approved by the Bristol-Myers Squibb (BMS) Institutional Animal Care and Use Committee. The animal care and use program at BMS is fully accredited by the Association for Assessment and Accreditation of Laboratory Animal Care International (AAALAC).

### Generation of HPK1 (MAP4K1) kinase KD mice

The targeting strategy is based on the NCBI transcript NM_008279_2. The targeting strategy allows the generation of a constitutive Knock-In of a point mutation (KI-PM) in the Map4k1 gene. Exon 1 contains the translation initiation codon. The K46M mutation was introduced into exon 2. The positive selection marker (Puromycin resistance—PuroR) was flanked by FRT sites and inserted into intron 5. The targeting vector was generated using BAC clones from the C57BL/6J RPCIB-731 BAC library and transfected into the Taconic Artemis C57BL/6N Tac ES cell line. Homologous recombinant clones were isolated using positive (PuroR) and negative (Thymidine kinase -Tk) selections. The constitutive KI-PM allele was obtained after in vivo Flp-mediated removal of the selection marker. This KI-PM allele was demonstrated to express the mutated Map4k1 K46M protein. The remaining recombination site is located in a non-conserved region of the genome.

### Tumor and immune response monitoring

1956 mouse sarcoma cells were maintained in DMEM based media. For tumor implantation, mice were given a subcutaneous injection of 0.1 ml cells at 1x10^7^/ml into the right flank with a 25 gauge needle on day 0. Tumors were allowed to grow to the pre-determined size window. The animals were then evenly distributed to various treatment and control groups with appropriate mean and median tumor values. Tumor implanted animals were sorted and randomized when tumors reached approximately 100mm^3^, typically on day 6 post implantation.

For all experiments, tumor volumes were measured using Vernier scale calipers, in two perpendicular directions, Length (L; the measurement of the longest axis) and Width (W; measurement of the shortest axis and perpendicular to L), using the formula: ½ (length x (width) e2). Groups were weighed and measured twice weekly until individual tumors or groups hit 1000 mm^3^. Mean, median and survival plots were generated to determine efficacy. If the mean tumor weight never reached target size, the treatment group was terminated or re-challenged when the remaining animals had stagnant tumor change for a period of >10 day of tumor volume doubling time (TVDT). Immune responses were monitored as described [17].

### T cell proliferation and cytokine analysis

For T cell proliferation, splenocytes or purified CD3+ T cells were stimulated with anti-CD3 and anti-CD28 for 72hr. The cells were pulsed during final 18hr culture prior to harvest with [3H]-thymidine followed by thymidine incorporation quantified by scintillation counter. Cytokine secretion was measured after 48hr post stimulation using AlphaLisa kits as suggested by the manufacturer (PerkinElmer, Akron, OH).

### Cytotoxic T lymphocyte (CTL) killing assay

1956 tumor cells were irradiated with 2000Gy. Splenocytes from tumor-bearing animals were depleted of red blood cells by treating them with RBC lysis buffer (eBioscience/ThermoFisher). Cytotoxicity against 1956 tumor cells was measured at variable effector to target (E:T) ratios after 4 h of co-culture in V-bottom 96-well plates, in the presence of 10 U/mL of IL-2. Percent lysis was calculated using the following formula: (%cytotoxicity = 100xE-min/max-min) where “min” is the minimum (target cells alone) and “max” is maximum (100%) lysis. The cytotoxicity was measured using lactate dehydrogenase kit (ThermoFisher, Waltham, MA).

#### Metabolomic profiling by liquid chromatography–mass spectrometry (LC-MS)

Samples from cell culture supernatant were subjected to protein precipitation by the addition of methanol containing 0.1% formic acid and stable-labeled internal standards. HILIC and RP LC-MS analyses were performed on a Nexera X2 LC-30AD (Shimadzu, Somerset, NJ) UHPLC system connected to an Exactive Plus (Thermo Fisher Scientific, Waltham, MA) mass spectrometer as described previously [18]. HILIC chromatographic separations were achieved by employing an Acquity BEH-NH2, 2.1x150 mm, 1.7 μm UHPLC column (Waters Corporation, Milford, MA) with mobile phases A (95:5 water: acetonitrile, 10 mM NH4OAc, 0.05% NH4OH) and B (acetonitrile, 0.05% NH4OH) at a flow rate of 300 μL/min with starting conditions of 95%B to 37%B at 3.5 min, hold for 4 min and down to starting conditions at 7 min, for a total run time of 11 min. RP chromatographic separations were achieved by employing an Acquity BEH C18, 2.1x150 mm, 1.7 μm UHPLC column (Waters Corporation, Milford, MA) with mobile phases A (water, 0.1% formic acid) and B (98:2 acetonitrile:water, 0.1% formic acid) at a flow rate of 600 μL/min with starting conditions of 100%A, to 80%A at 3 min, 40%A at 4 min and 100%B by 7 min and after a 2 min hold, down to starting conditions at 9 min, for a total run time of 11 min. Both HILIC and RP LC-MS data were collected in positive and negative polarities by separate injections at 35,000 resolution and an expected mass accuracy of 5 ppm.

Data analysis was conducted using in-house developed Expedient Data Mining (EDM) software as described previously [19]. Mean intensity values for each group of tumor cell line supernatants were compared to mean intensity values for control media samples. p-values were calculated as a pair-wise comparison for a two-tailed distribution, using Student’s t-test (Excel statistics package, Microsoft).

### PGE2 quantification by LC-MS

PGE2, PGE2-d9, PGD2, 15-keto-PGF2α, PGA2, PGB2, and PGJ2 analytical standards were purchased from Cayman Chemical (Ann Arbor, MI). All solvents used for LC-MS were HPLC Plus grade from Sigma Aldrich Corp. (St. Louis, MO).

Cell culture supernatants along with relevant standards were undergone extraction and samples were analyzed by LC-MS using a Shimadzu Nexera UHPLC (Shimadzu North America, Columbia, MD) interfaced to a Q-Exactive Plus ion trap mass spectrometer with a HESI source (ThermoFisher Scientific, San Jose, CA). Chromatographic separations were achieved by employing a 2.1 x 100 mm, 1.8 μm, Acquity HSS T3 column (Waters, Milford, MA) with gradient elution at 0.45 mL/min [20]. PGE2 was quantified against PGE2-d9 internal standard. The standard curve was generated using a constant concentration of PGE2-d9 (50 pg on column) and variable concentrations of PGE2 (2 pg to 10 ng on column). Peak areas for LC-MS quantification of PGE2 and PGE2-d9 were calculated using Xcalibur QuanBrowser software (ThermoFisher Scientific, San Jose, CA). The coefficient of determination for the standard curve (R2) was 0.9996.

### Statistical analysis

Data were plotted as the mean ± SEM. Student t test, one-way or two-way ANOVA was carried out using GraphPad Prism (GraphPad Software, La Jolla, CA) and statistical significance was considered meaningful at p<0.05.

## Results

### Generation and immune phenotyping of HPK1 KD mice

**Fig 1:**
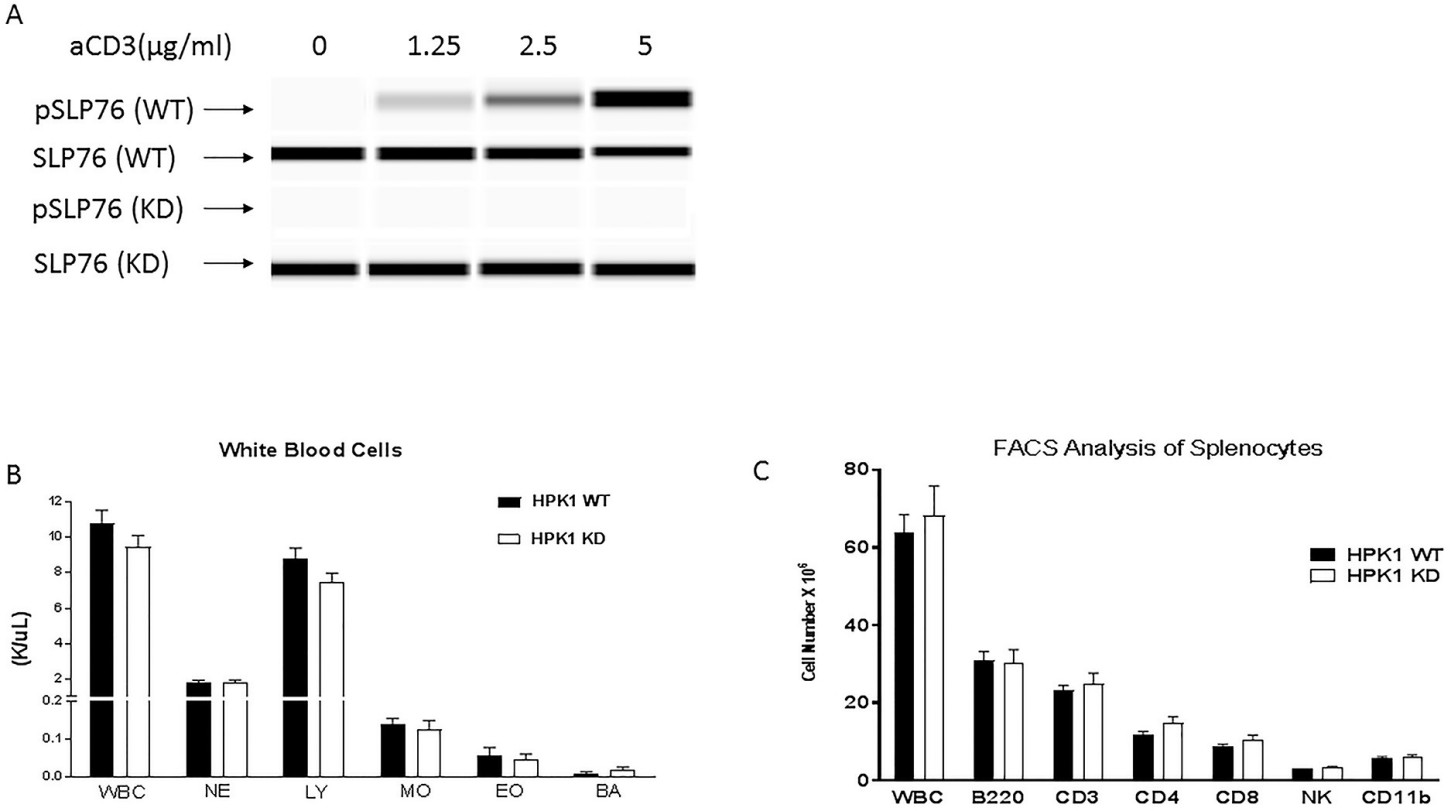
Characterization of HPK1 KD mice. **A**. Abolished phosphorylation on Ser376 of SLP76 by HPK1 KD. Splenocytes from HPK1 WT and KD mice were stimulated with anti-CD3 and SLP76 phosphorylation was determined by Western blot. **B**. Characterization of immune cell populations in peripheral blood. **C**. Evaluation of immune cell populations with splenocytes isolated from HPK1 WT and KD mice. N = 5. WBC: white blood cells; NE: neutrophils; LY: lymphocytes; MO: monocytes, EO: eosinophils; BA: basophils.

The point mutation K46M was constitutively introduced into the kinase domain of the mouse HPK1 gene, resulting in a kinase-inactive HPK1. The kinase dead knock-in mice (KD) were generated on a C57/Bl6 background (S1 Fig). HPK1 KD mice were born at the expected mendelian ratio and were fertile. The inactivation of HPK1 kinase activity in the KD mice was further confirmed by the lack of serine 376 phosphorylation of SLP (SLP76), a substrate of HPK1, upon T cell receptor (TCR) engagement (Fig 1A). Phenotypic characterization of these mice was subsequently conducted including hematology, flow cytometry analysis (FACS) of splenocytes, long term body weight monitoring, health assessments and organ weights in naïve animals. No notable differences were observed for body or organ weights and general health abnormality with inactivation of HPK1 kinase (S1 Table). White blood cell, red blood cell and platelet counts did not differ significantly between the KD and WT mice (Fig 1B and S5 Fig). FACS analysis splenocytes from 11 week-old female HPK1 WT and KD mice (5 mice of each genotype) was performed. The splenocytes were stained with anti-B220, anti-CD3, anti-CD4, anti-CD8, anti-pan-NK, and anti-CD11b monoclonal antibodies followed by measurement via FACS to evaluate various immune cell populations. No significant differences were detected in the FACS analysis for splenocytes from HPK1 WT and KD mice (Fig 1C), suggesting that kinase activity of HPK1 is not essential for leukocyte development and maintenance in homeostatic environment.

### Proliferation and cytokine release in HPK1 KD T cells

**Fig 2:**
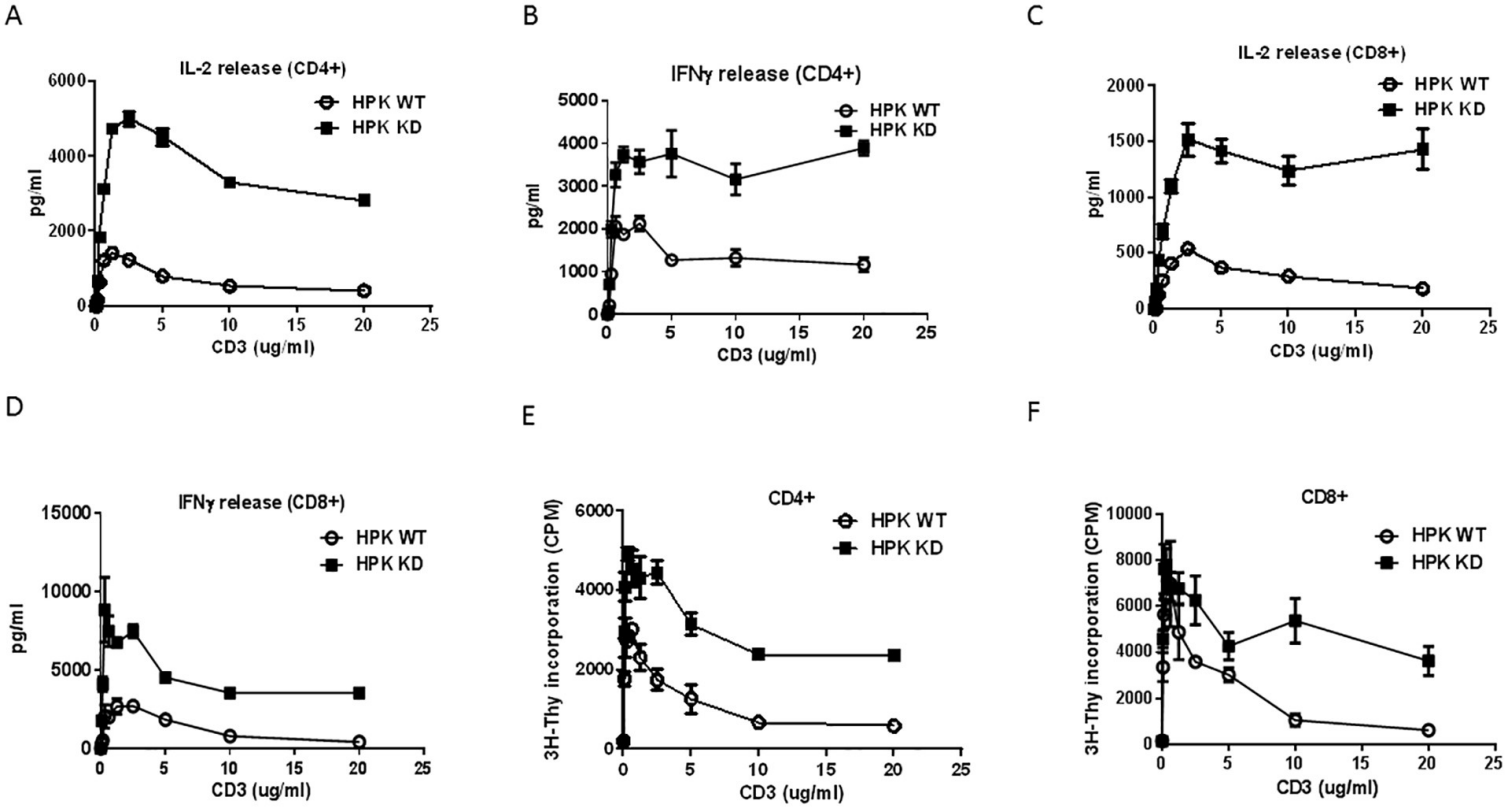
Assessment of CD4^+^ and CD8^+^ T cells from HPK1 WT and KD mice. **A-B**. IL-2 and IFNγ secretion from CD4^+^ T cells after stimulation. CD4^+^ T cells were isolated and purified from splenocytes. IL-2 and IFNγ secretion were measured after 48hr of anti-CD3 and anti-CD28 stimulation. **C-D**. IL-2 and IFNγ release from CD8^+^ T cells upon stimulation. CD8^+^ T cells were isolated and purified from splenocytes. IL-2 and IFNγ secretion were measured after 48hr of anti-CD3 and anti-CD28 stimulation. **E-F**. Proliferation of CD4^+^ and CD8^+^ T cells after 72hr stimulation with anti-CD3 and anti-CD28. [3H]-Thymidine incorporation was utilized to monitor the proliferation. The data shown above are representative graphs from 3 independent studies.

HPK1 was demonstrated to be involved in T cell activation via modulation of TCR mediated signaling and HPK1 KO T cells were hyper-proliferative in response to stimulation with agonistic antibody to CD3. To evaluate the role of HPK1 kinase activity in mediating TCR-mediated T cell proliferation and cytokine release, CD4 and CD8 positive T cells were purified from splenocytes of HPK1 WT and KD mice. The cells were then stimulated with plate-bound anti-CD3 and soluble anti-CD28 agonistic antibodies. The supernatants from the cell culture were collected after 48hr stimulation. The release of IL-2 and IFNγ was measured. The inactivation of HPK1 kinase led to significant enhancement of IL-2 and IFNγ release by both CD4+ and CD8+ T cells (Fig 2A–2D). Further analysis of T cell proliferation by [^3^H]-thymidine incorporation revealed a hyper-proliferative response upon inactivation of HPK1 kinase (Fig 2E–2F). These results are consistent with the observation made with HPK1 KO T cells and suggest that the kinase activity of HPK1 is critical in its immune modulatory functions as a negative regulator.

### Augmented cytokine release and T cell proliferation in HPK1 KD mice upon in vivo challenge with anti-CD3 or ovalbumin (OVA)

To investigate the correlation of enhanced in vitro proliferation of HPK1 KD T cells with in vivo activation of HPK1 KD T cells, agonistic mouse CD3 antibody was injected via the tail vein and changes of cytokine levels in plasma were evaluated after 1.5hr of anti-CD3 administration. Significant enhancement of serum cytokine and chemokine levels was observed including IL-2, IFNγ, IL-10, IL-6 and CXCL10 (S1 Fig). Furthermore, BrdU incorporation into dividing cells was utilized to measure T cell proliferation in vivo after immunization with OVA. OVA-induced T cell proliferation determined by BrdU incorporation revealed a significant increase in the number of proliferating CD45^+^, CD4^+^, CD3^+^ cells in both the spleens and lymph nodes from KD versus WT mice (S1 Fig). To determine whether HPK1 kinase activity influences humoral responses, we measured antigen-specific antibody production in immunized WT and KD mice. OVA-induced antibody production was detected at significantly higher titers of IgG1 and IgG2b in the sera of KD mice relative to the WT controls; IgG2a was also elevated in KD serum samples but was not statistically significant after secondary immunization (S2 Fig). In addition, the keyhole limpet hemocyanin (KLH) challenge resulted in significantly higher IgG titers in serum samples from KD mice compared to the WT controls with similar levels of IgM (S2 Fig). These results indicate that lack of HPK1 kinase activity resulted in a more vigorous T cell–dependent humoral response in immunized mice.

### Enhanced cytolytic activities in NK cells and dendritic cell function with HPK1 KD

Expression of HPK1 was observed in natural killer (NK) cells (S3 Fig). To investigate the role of HPK1 in NK cells, the cytolytic activity was measured using HPK1 WT and KD NK cells. NK sensitive murine lymphoma cells (YAC-1) were used as the targets. HPK1 KD NK cells exhibited a significant increase (p = 0.0058) in the percent of cytotoxic activity compared to the WT controls (S3 Fig). HPK1 KO was demonstrated to improve DC functions [4]. To investigate the potential improvement of DC function, we utilized bone marrow derived DCs (BMDCs) from HPK1 WT and KD mice to assess their antigen presentation capacity. The responder cells were CD8^+^ T cells from OVA specific TCR transgenic mice (OT1). The responder cells were co-cultured with BMDC pulsed with Ova-derived peptides for 3 days. Significant enhancement of OT1 cell proliferation was observed with BMDCs from HPK1 KD mice (S3 Fig).

### HPK1 KD T cells are resistant to immune suppression exerted by PGE2 and adenosine

**Fig 3:**
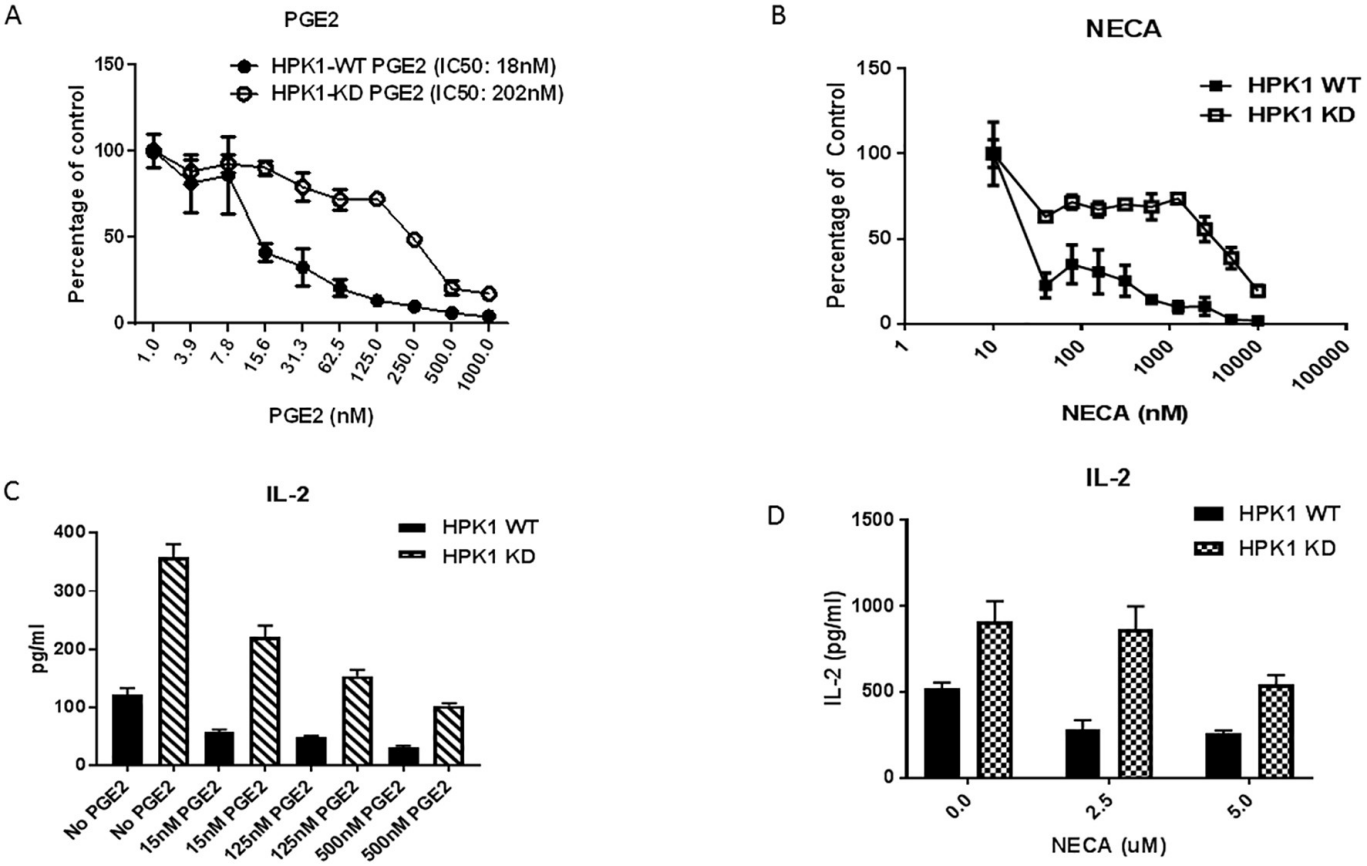
Resistance of HPK1 KD CD3^+^ T cells in the presence of immune suppressive factors. **A**. Proliferation of CD3^+^ T cells under various concentrations of PGE2. CD3^+^ T cells were isolated from HPK1 WT and KD spleen. The T cells were stimulated with anti-CD3 and anti-CD28 in the presence of PGE2 for 72hr. **B**. Proliferation of CD3^+^ T cells under various concentrations of stable adenosine analogue, NECA. The T cells were stimulated with anti-CD3 and anti-CD28 in the presence of NECA for 72hr and cell proliferation was measured by [3H]-thymidine incorporation. **C**. Resistance of PGE2 suppression of IL-2 release by HPK1 KD. **D**. Resistance of NECA suppression of IL-2 by HPK1 KD. All studies were repeated 3 times. The data shown here are representative graphs from 3 independent studies.

Immune suppressive factors are critical components eliciting resistance to tumor immune therapy. The prostaglandin PGE2 in the tumor is a key mediator of resistance to immunotherapies. Another immune suppressive factor, adenosine, impairs the cytotoxic anti-tumor immune response, while supporting proliferation and polarization of immunosuppressive cells and neovascularization to accelerate tumor growth. To investigate the role of HPK1 kinase activity in mediating the T cell response to PGE2 and adenosine, we isolated the splenocytes from HPK1 WT and KD mice and stimulated these cells with anti-CD3 in the presence of different concentrations of PGE2 and a stable adenosine analogue, 5′-(n-ethylcarboxamido) adenosine (NECA). The proliferation of the splenocytes was measured after 72 hr of stimulation via [^3^H]-thymidine incorporation for the last 18hr of cell culture. PGE2 and adenosine exhibited inhibitory effects on splenocyte proliferation in a concentration dependent manner. The inactivation of HPK1 kinase led to significant resistance to the impairment of splenocyte proliferation by PGE2 and NECA (Fig 3A–3B). The suppression of IL-2 release by PGE2 and adenosine was also significantly weakened with HPK1 KD (Fig 3C–3D), suggesting that the kinase activity of HPK1 plays a critical role in attenuating the suppressive functions of PGE2 and adenosine.

### Inactivation of HPK1 kinase improves anti-tumor efficacy in a mouse sarcoma model

**Fig 4:**
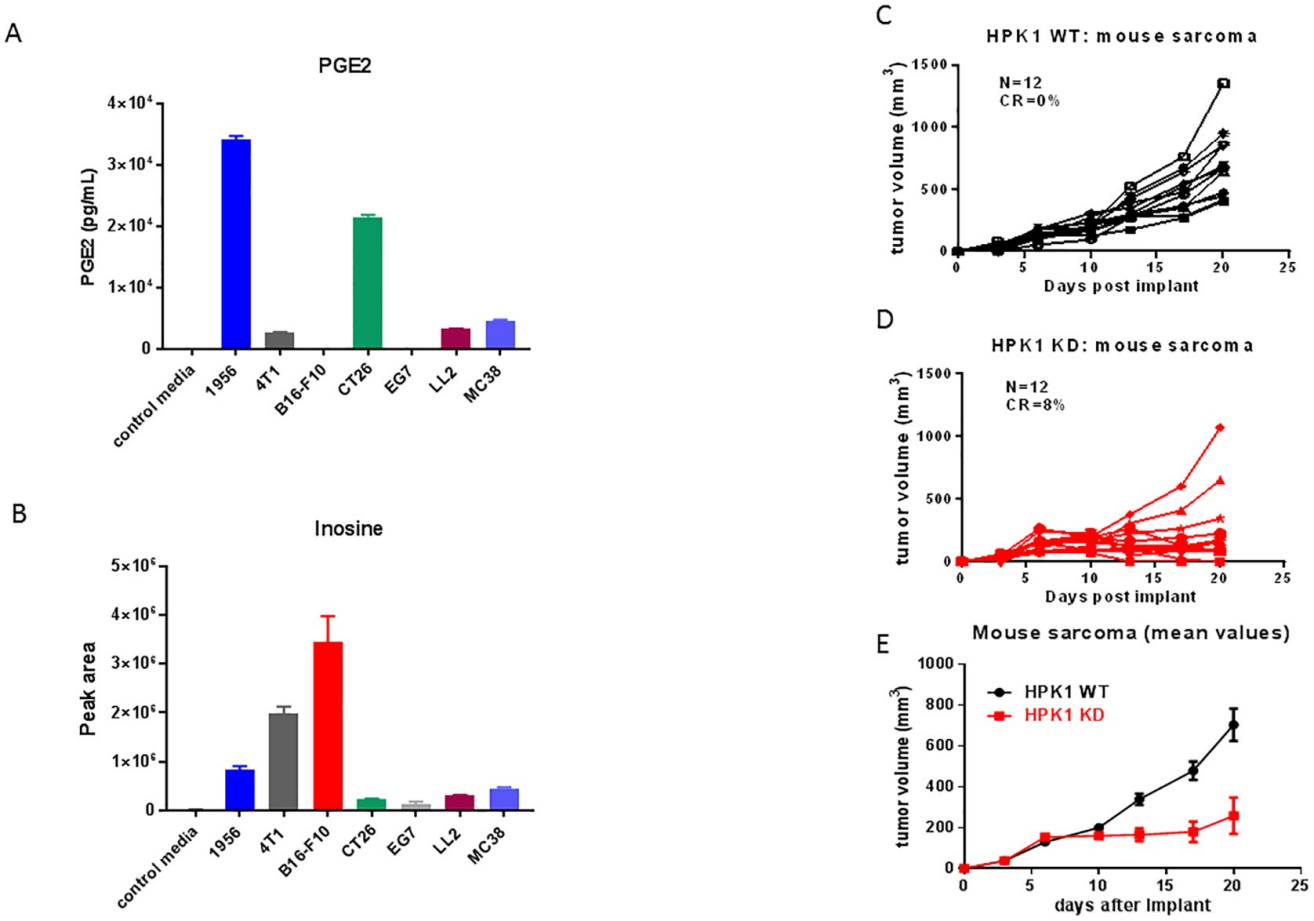
The effect of HPK1 KD on anti-tumor efficacy in mouse sarcoma model. **A**. Production of PGE2 in various mouse tumor cells. PGE2 levels from the supernatants of cultured tumor cells were measured using LC-MS. **B**. Release of inosine, as the surrogate of adenosine, in various mouse tumor cells. Inosine levels from the supernatants of cultured tumor cells were measured using LC-MS. **C**. Tumor progression monitored over time with individual HPK1 WT mouse. **D**. Tumor progression measured over time with individual HPK1 KD mouse. **E**. The comparison of mean tumor volumes over 21 days for HPK1 WT and KD mice implanted with 1956 sarcoma cells. n = 10 per group.

There is significantly enhanced production of PGE2 and adenosine in various types of tumor cells. To further determine the levels of PGE2 and adenosine generated by various murine tumors, we employed LC-MS technology to quantify PGE2 and adenosine levels across different tumor types. Inosine was utilized as a surrogate for adenosine in this study due to the metabolically labile nature of adenosine. PGE2 levels were significantly higher in murine sarcoma (1956) and murine colon carcinoma (CT26) cells, and also elevated in other murine tumor cell lines (MC38, LL2, 4T1) (Fig 4A). Augmented inosine levels were observed in murine melanoma (B16-F10), mammary (4T1) and sarcoma (1956) cells, as well as other cell lines (Fig 4B). These data suggested the potential immune suppressive effects by PGE2 and adenosine in the corresponding tumors. With high levels of release of both PGE2 and inosine (adenosine) from 1956 sarcoma cells, we selected this tumor model to assess the potential anti-tumor benefit afforded by the inactivation of HPK1 kinase activity. 1956 mouse sarcoma cells were implanted into syngeneic HPK1 WT and KD C57/BL6 mice and tumor progression and survival rate were monitored over time. A significant slowing of tumor growth was observed in HPK KD mice (Fig 4C–4E). These results suggest that inactivation of HPK1 kinase contributes to the improvement of anti-tumor immune response in a tumor model containing relevant immune suppressive factors, e.g. PGE2 and adenosine.

### Immune modulatory functions of HPK1 in situ during anti-tumor response

**Fig 5:**
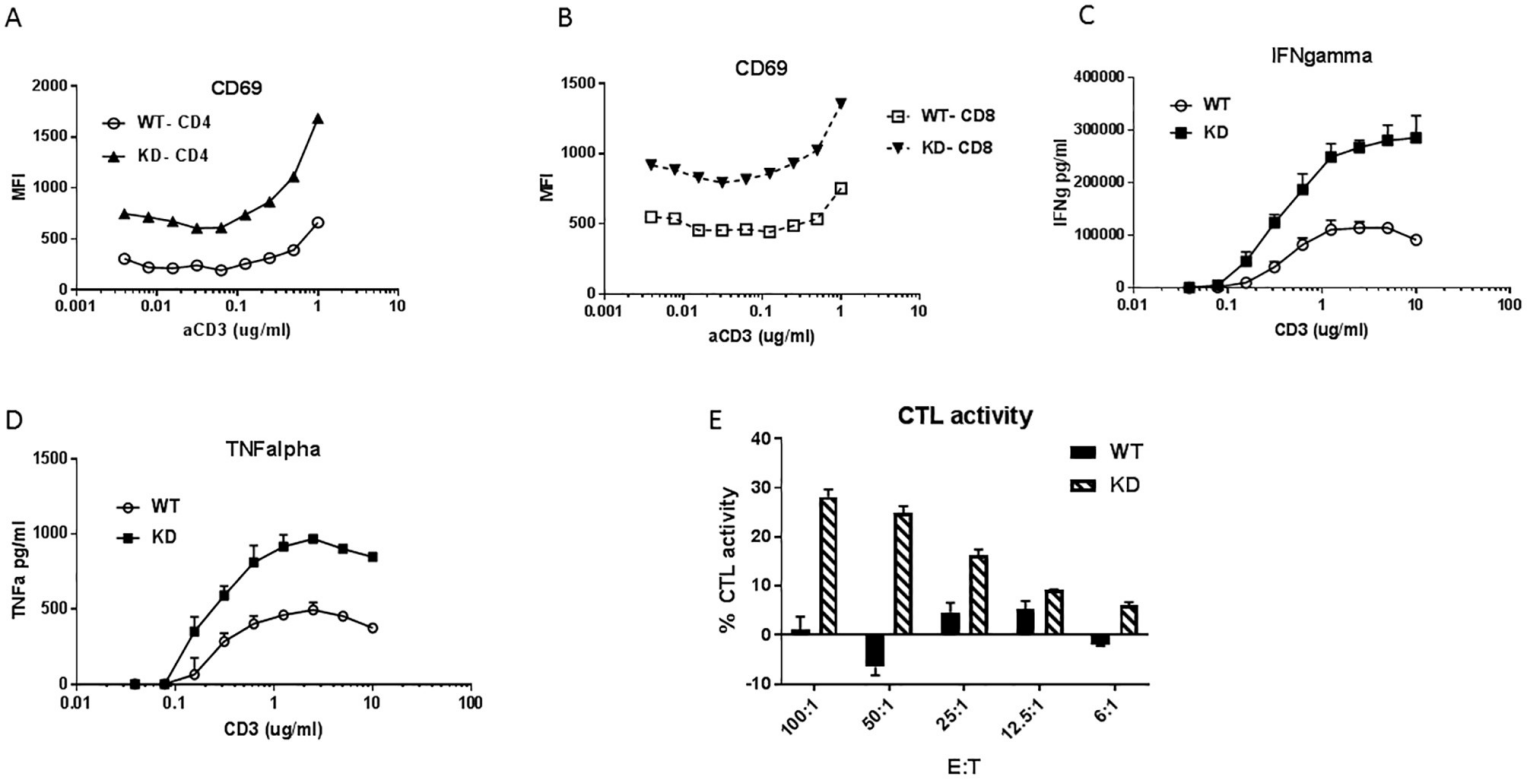
Ex Vivo functional evaluation of HPK1 WT and KD splenocytes after tumor cell priming. Splenocytes were isolated after 30-day of tumor implantation in HPK1 WT and KD mice. **A**. Surface levels of CD69 on CD4^+^ T cells under ex vivo stimulation with anti-CD3 for 3hr. CD69 levels were determined with FACS analysis. **B**. Surface levels of CD69 on CD8^+^ T cells after stimulation with anti-CD3 for 3hr. **C**. IFNγ release with tumor-primed splenocytes after 48hr ex vivo stimulation with anti-CD3. **D**. TNFα release with tumor-primed splenocytes after 48hr ex vivo stimulation with anti-CD3. **E**. Cytolytic activities of HPK1 WT and KD splenocytes (E: effector cells) against syngeneic tumor cells (T: target cells) with various ratios of E: T. The cytotoxicity was measured via the release of lactate dehydrogenase to the supernatants of the co-culture. The data are the representative graphs of 5 individual mice from WT and KD groups.

To further demonstrate the impact of HPK1 KD on immune cell functions in the context of anti-tumor immune response, we isolated splenocytes from HPK1 WT and KD mice implanted with 1956 sarcoma for 30 days. These splenocytes were subsequently stimulated with anti-CD3 ex vivo for 3 hr. Cell surface levels of CD69 were analyzed with FACS. Significantly higher CD69 levels, in both CD4^+^ and CD8^+^ cell populations, were observed in the HPK1 KD group in comparison with WT group (Fig 5A–5B), indicating that HPK1 KD leads to augmentation of T cell activation in the tumor bearing mice. Secretion of IFNγ and TNFα was also evaluated with these splenocytes after 48hr ex vivo stimulation with anti-CD3. The magnitude of the cytokine release was markedly higher in HPK1 KD groups (Fig 5C–5D), with over 30 fold higher levels of IFNγ in the tumor bearing mice compared with naïve mice (Fig 2D and Fig 5C). Furthermore, to investigate the ability of tumor antigen experienced HPK1 KD T cells to elicit an anti-tumor response, splenocytes from WT and HPK1 KD tumor bearing mice were cultured with irradiated 1956 tumor at different effector and target cell ratios for 4hr followed by the measurement of tumor cell lysis using LDH release. The HPK1 KD T cells, in comparison with HPK1 WT T cells, exhibited remarkably higher cytolytic activities against the previously experienced syngeneic tumors (Fig 5E), suggesting that HPK1 KD could improve the antigen recall response of CD8^+^ T cells.

### Improved anti-tumor immune phenotypes in TME with HPK1 KD

**Fig 6:**
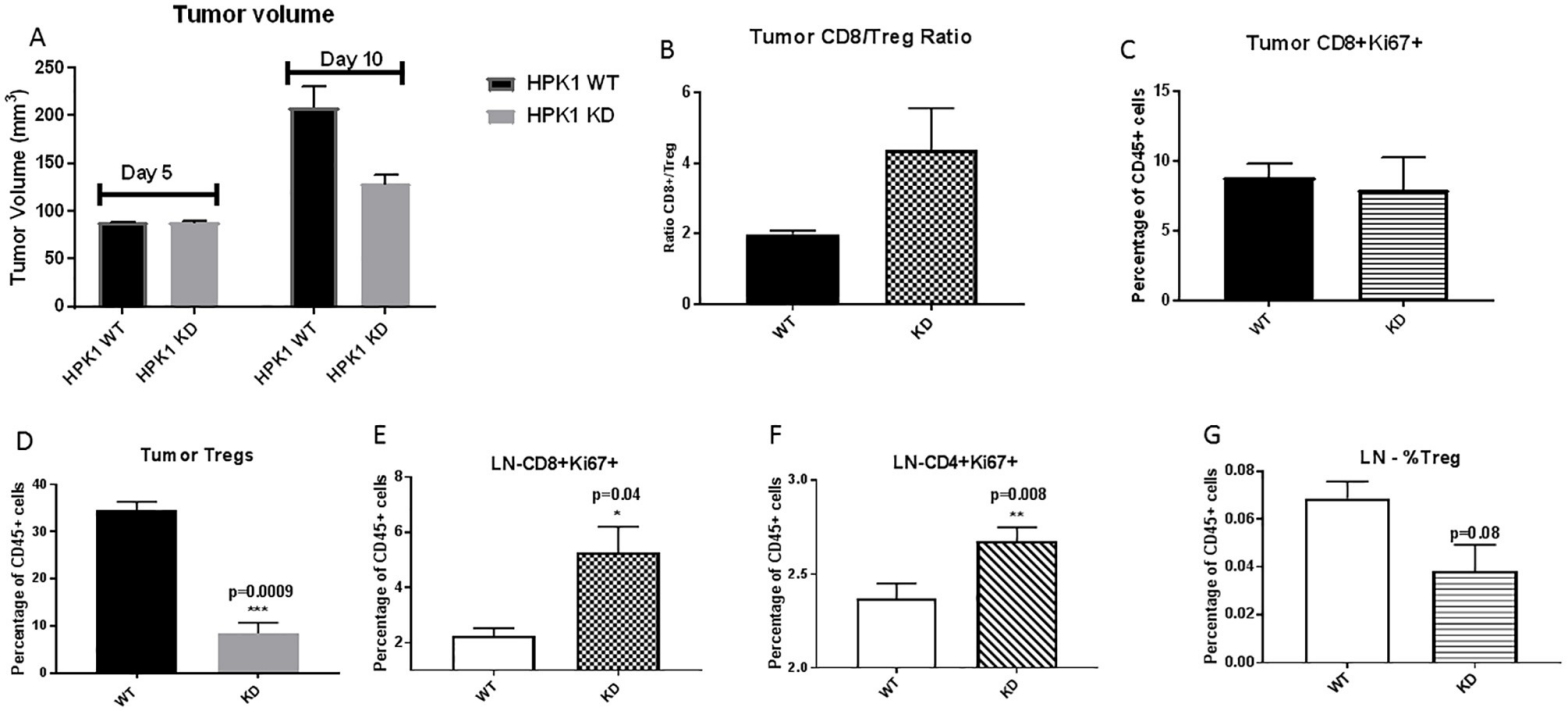
Analysis of tumor infiltrated lymphocytes (TIL) in mouse sarcoma model under HPK1 WT and KD background. TILs were isolated after 10 days of tumor implantation. **A**. Tumor progression at day 5 and day 10 post tumor implantation. **B**. Ratio of CD8^+^/Treg in tumors from WT and KD mice. CD45^+^ TILs were analyzed by FACS. **C**. The percentage of CD8^+^Ki67^+^ T cells from tumors determined by FACS. **D**. The percentage of regulatory T cells in tumors as defined by CD4^+^CD25^hi^FOXP3^+^. **E**. The percentage of CD8^+^Ki67^+^ T cells from dLN. Single cell suspension was generated from tumor dLN and analyzed by FACS. **F**. The percentage of CD4^+^Ki67^+^ T cells from dLN. **G**. The percentage of regulatory T cells from tumor dLN as defined by CD4^+^CD25hiFOXP3^+^. N = 15 per group.

Effective immune cell infiltration is essential to elicit the attack against tumor cells. To evaluate the critical role of HPK1 kinase activity in mediating the improvement of anti-tumor immune cell populations in the tumor microenvironment (TME), we dissociated the tumors and draining lymph nodes (dLN) 10-day post tumor implantation. Immunophenotyping was conducted to determine the key changes in immune cell populations in the TME and dLN in the context of WT and KD HPK1. A modest reduction of tumor volume was observed in HPK1 KD mice post 10-day tumor implantation (Fig 6A). Further analysis revealed significant reduction of regulatory T cells, an increased ratio of CD8^+^ to regulatory T cells in the TME of HPK1 KD mice, whereas the percentage of CD8^+^ Ki67^+^ T cell populations remained similar between WT and KD groups (Fig 6B–6D). The dLN analysis from HPK1 KD mice demonstrated a markedly higher percentage of CD4^+^Ki67^+^ and CD8^+^Ki67^+^ T cells as well as reduction of the regulatory T cell population (Fig 6E–6G). Collectively, these data suggest that HPK1 kinase activity plays an important role in enabling anti-tumor immune responses, potentially via the improved CD8^+^T cell functions and reduction of regulatory T cells in the TME. Importantly, under HPK1 KD conditions, the dLN appear to be better orchestrated to generate a more robust anti-tumor immune reservoir as demonstrated by the augmented levels of CD4^+^Ki67^+^ and CD8^+^Ki67^+^ T cells.

### Evaluation of immune cell profiles in TME

**Fig 7:**
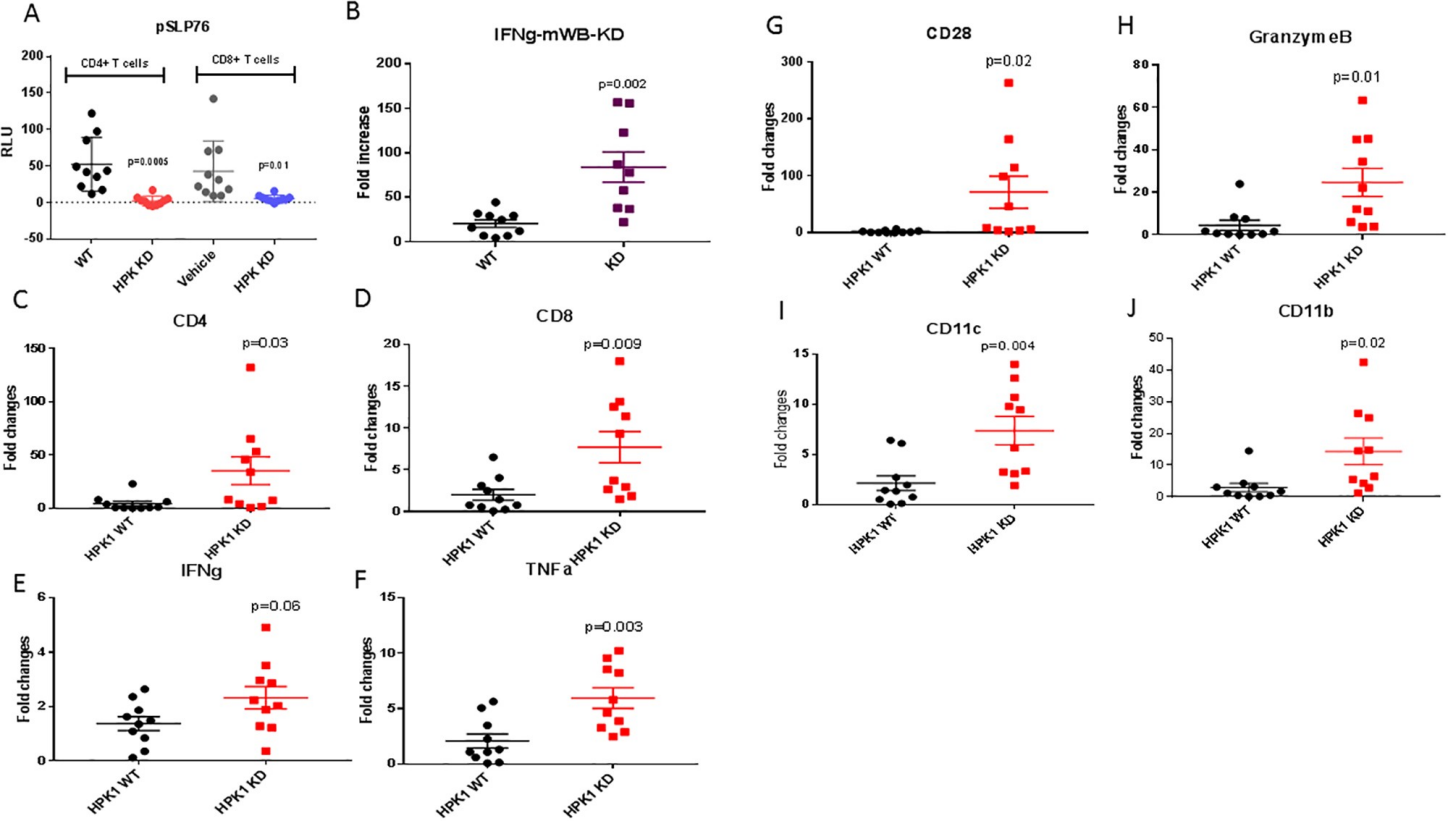
Immunophenotyping of whole blood and tumors from HPK1 WT and KD mice. Blood and tumor samples were collected 10 days after tumor implantation. **A**. Inhibition of TCR activation induced Ser376 phosphorylation of SLP76 by HPK1 KD. The blood samples from tumor-bearing HPK1 WT and KD mice were analyzed after ex vivo stimulation with anti-CD3 and anti CD28 for 15min. pSLP76 levels were determined for CD4^+^ and CD8^+^ T cells from peripheral blood. **B**. IFNγ levels from peripheral blood T cells after ex vivo stimulation with anti-CD3 and anti-CD28 for 3hr and analyzed by qPCR. qPCR analysis was conducted using RNA isolated from tumors. CD4 (**C**), CD8 (**D**), IFNγ (**E**), TNFα (**F**), CD28 (**G**), GranzymeB (**H**), CD11c (**I**), CD11b (**J**). N = 10 per group.

To further capture the immune modulatory signatures of tumors in HPK1 KD mice, we conducted a study in the 1956 sarcoma model 5 days after tumor implantation. At the end of the study, modulation of TCR activation-induced pSLP76 and IFNγ was assessed in mouse whole blood following ex vivo re-stimulation with anti-CD3 and anti-CD28. In comparison with HPK1 WT mice, a significant reduction of pSLP76 was observed in both CD4^+^ and CD8^+^ T cells in the peripheral blood of the HPK1 KD group (Fig 7A), with markedly higher IFNγ messenger levels in HPK1 KD group (Fig 7B). Quantitative PCR analysis was conducted using RNA from tumor homogenates. Tumor samples from HPK1 KD mice demonstrated significantly upregulated key immune cell markers involved in mediating anti-tumor immunity, including CD4, CD8, IFNγ, TNFα, Granzyme B, CD28, CD11c and CD11b (Fig 7C–7J). These results suggest that the lack of HPK1 kinase activity leads to a greater activated immune cell presence in the TME. The immune signatures of tumor draining lymph nodes were further interrogated using nanostring analysis. Under tumor challenge conditions, significant immune modulatory effects were observed in HPK1 KD group compared with WT group (S4 Fig). The results revealed that various immune cell types and pathways were affected in HPK1 KD mice, including T effector cells, dendritic cells, regulatory T cells, natural killer cells and neutrophils. Notably, HPK1 KD mice showed markedly increased pro-inflammatory pathways in response to priming with tumor antigens as shown by the enhanced related gene expression, e.g. S100A8, GZMB, NKp44, CXCL14, CX3CL1, CD1d2, KLRG1, CD11c, NLRP3 and MUC1. On the other hand, genes related to T helper 2 and regulatory T cells were downregulated, e.g. TGFß2, Arg1, CD30, AIRE, PTGS-2, EGR-2, CXCR4, IL-4 and CCL-17 (S4 Fig). Further analysis indicates that inactivation of HPK1 kinase improved pathways related to chemokine/receptor signaling, adaptive immune responses and dendritic functions in dLN with down-regulation of apoptotic network (S4 Fig). Together, these data strongly suggest that the kinase activity of HPK1 has a profound impact on the priming of anti-tumor immune responses in the dLN and further support that HPK1 serves as a negative regulator of the immune network in a tumor setting.

## Discussion

The desired outcome of cancer immunotherapy is to increase response rate, durability and overcome resistance. Both tumor cell intrinsic and extrinsic factors contribute to the resistance mechanisms [8]. It is valuable to identify therapeutic targets that could simultaneously improve T cell function, tumor antigen presentation and combat the immunosuppressive tumor microenvironment. Through the studies in KO mice, the beneficial effects of HPK1 deletion on the improvement of TCR signaling, antigen presentation ability and resistance to PGE2 immunosuppression have been well documented [7, 16, 21–30]. With its predominant expression in hematopoietic cell linages and its multilateral impact on key immune cell types involved in anti-tumor action, pharmacological blockade of HPK1 may provide superior anti-tumor potential [7]. Previous studies were conducted primarily with HPK1 WT and KO mice. However, it is more pharmacologically feasible to disrupt HPK1 activity with a small molecule kinase inhibitor. The current study has demonstrated that the kinase activity of HPK1, and not the potential scaffolding function of the protein, plays a key role in the anti-tumor immune response, consistent with previous findings of HPK1 as a negative regulator of TCR-induced T-cell activation and as one of the molecules that maintain peripheral tolerance as revealed with HPK1 KO models [5].

We demonstrated that HPK1 KD exhibits significant impact on both CD4^+^ and CD8^+^ T effector cells with enhanced cytokine secretion and proliferation under in vitro and in vivo settings, as well as under polyclonal stimulation conditions with anti-CD3 or an antigen specific manner with OVA administration. These positive effects could play a pivotal role to boost immune response in the context of the TME. Besides the effect of HPK1 KD on CD8^+^ T cells, there was also significant impact on CD4^+^ T cells with HPK1 KD, suggesting a positive role of HPK1 KD on T helper cells. In the context of dysfunctional CD4^+^ T helper cells, the “helpless” tumor specific CD8^+^ T cells could ultimately develop functional deficits with impaired tumor-specific CD8+memory T-cell responses [31–35]. The CD4^+^ T cells with HPK1 KD could improve the priming phase of CD8^+^ T-cell activation and the differentiation of CD8^+^ effector cells into memory cells, via direct cell-cell interaction and/or IL-2 provision. In addition, CD4^+^ T cells also orchestrated dendritic cells (DCs) to activate CD8^+^ T cells either by cross-presenting tumor antigens to CD8^+^ T cells or by inducing the production of IL-2. Strikingly, our current study revealed a marked augmentation of CD4^+^Ki67^+^ and CD8^+^Ki67^+^ T cells in tumor dLN in HPK1 KD mice, further support the positive interplay between CD4^+^ and CD8^+^ T cells upon inactivation of HPK1 kinase in tumor bearing mice. A recent report, with SLP76-S376A mutated T cells, indicates that HPK1 is also involved in mediating fine tuning of helper T cell responses with increased Th1-type and decreased Th2-type cytokine production [36], suggesting a role for HPK1 as a negative regulator of proinflammatory Th1 cells. Consistently, our data here demonstrated a significant enhanced IFNγ secretion with CD4^+^ T cells from HPK1 KD mice. IFN-γ can up-regulate MHC molecules to increase the number of peptide-MHC complexes and alter the antigen-processing machinery leading to enhanced tumor recognition as well as greater tumor cell lysis [37]. In supporting the critical role of HPK1 for the generation and maintenance of effective cytotoxic and memory CD8+ T cells by facilitating the optimal expansion and effector function of CD8^+^ T cells, we observed that HPK1 KD CD8^+^ T cells displayed greater activation status with higher CD69 expression upon being primed with tumor cells, along with markedly higher levels of IFNγ and TNFα release with ex vivo re-stimulation. Moreover, a significantly better recall response was demonstrated with HPK1 KD splenocytes upon ex vivo re-challenge with syngeneic tumor cells.

In the case of naturally acquired resistance during cancer immunotherapy, the resistance may mostly reflect the mechanisms that interfere with T-cell activity within the TME. Elevated activities of PGE2 and adenosine pathways have been shown to play an important role in the establishment of a long-lasting immunosuppressive environment in different types of tumors. These immunosuppressive factors are currently viewed as a significant barrier to the effectiveness of immune therapies and have become important therapeutic targets in cancer. Tumor cells, via overexpressing COX2 and elevating PGE2 production, favor the development of a Treg phenotype in human and mouse CD4^+^ T cells. Aberrant or increased expression of COX-2 and its major metabolite, PGE2, are found in several malignancies including non small cell lung carcinoma (NSCLC) in which they promote metastatic development [38]. While COX-2/ PGE2 and TGF-β are implicated in tumorigenesis via different mechanisms [38–39], both are capable of generating peripherally induced Treg [40–43]. Treg are operative in the inhibition of local antitumor immunity and promotion of cancer progression. On the other hand, adenosine is one of the major immunosuppressive factors utilized by Treg cells for reducing responses to self, regulating tolerance to tissue grafts or cancer, and preventing autoimmune diseases [44–45]. Adenosine and PGE2 thereby cooperatively diminishes lymphocyte activities [46]. The TME enriched with adenosine and PGE2 could favor the expansion of more immunosuppressive COX-2^+^ and ectonucleotidase+ T cells. Our study revealed that HPK1 KD T cells were over 10 fold more resistant to PGE2 challenge, which is consistent with previous findings with HPK1 KO T cells [16, 26]. Aberrantly high levels of adenosine, generated by hypoxic conditions or apoptotic cells in the TME, are involved in establishing an immunosuppressive environment and abolishing PD-L1-blockade-mediated antitumor T cell immunity [47–48]. Importantly, HPK1 KD T cells were demonstrated to display robust resistance to the immune suppressive action of NECA, the stable adenosine analog. Our data strongly support that the inhibition of the kinase activity of HPK1 could facilitate the maintenance of effective anti-tumor immune responses and overcome immune resistance mediated by immune suppressive factors. While PGE2 was reported to activate HPK1 in a TCR independent, cAMP/PKA dependent manner in T cells [25], the mechanistic interplay between the adenosine pathway and HPK1 remains to be further investigated. To further investigate the anti-tumor efficacy of HPK1 KD in a type of tumor releasing high levels of PGE2 and adenosine, we employed 1956 sarcoma as our model of study. In HPK1 KD mice, tumor progression was significantly retarded and an enhanced ratio of CD8+/Treg in the TME was observed. This improved anti-tumor immune signature was also demonstrated by qPCR analysis using RNA from total tumor homogenates. Our qPCR results revealed enhanced overall immune competence (i.e. the ability to elicit a polyfunctional T-cell response) in the TME of HPK1 KD mice, as reflected by the boost in T effector cell functions, superior antigen presentation and a reduction of the suppressive T regulatory cell population. HPK1 was reported to be a critical component in TGFß induced activation of JNK1 [49], a pathway involved in promoting the differentiation of Foxp3^+^ Treg induced by TGF-β [50]. A significant reduction of the Treg population in the TME of HPK1 KD mice was observed, potentially via intervention of the TGFß-HPK1-JNK and/or PGE2-HPK1 cascades. Additionally, loss of HPK1 kinase activity is likely to re-shape the immune cell repertoire/balance via modulating the plasticity of Tregs and promoting the conversion of Treg into T effector cells. Consistently, previous study revealed that HPK1 KO Tregs had weaker suppressive acitivity and produced significantly higher levels of IL-2 in response to TCR engagement [51]. Treg cells are well documented to be a potential therapeutic target in cancer therapy [52]. Depletion of Treg cells in the TME would shift the balance from immune suppression to immune activation towards tumor cells. In constant Treg supportive TME with Treg populations being constantly replenished by conversion of conventional CD4^+^CD25^-^ T cells into CD4^+^CD25^+^ Treg upon exposure to TGF-β or PGE2 [40–41, 53–54], inhibition of HPK1 could combat Treg suppression via several venues as demonstrated by our studies here. Additional studies are needed to further dissect HPK1-mediated key nodes in various cancer types. During the review process of this manuscript, an independent study with HPK1 KD mice was reported and the data revealed kinase activity of HPK1 as the key driver of T cell inhibition in a syngeneic murine model of adenocarcinoma (MC38), which is supportive and complementary to our findings in this study [55]. Together, our data here demonstrate that blockade of HPK1 kinase activity could simultaneously address several key challenging factors in current immunotherapy (e.g. immune suppressive TME/immune evasion, dysfunctional T effector cells and NK cells, impaired tumor antigen presentation, primary and adaptive immune resistance), and it is likely that HPK1 inhibition will synergize with modulators of immune checkpoints as well as targets related to the PGE2 and adenosine pathways.

## Supporting information

S1 Fig Generation of HPK1 KD and in vivo challenge with anti-CD3 and OVA in HPK1 WT and KD mice. **A**. Scheme for the generation of HPK1 KD construct. A constitutive knock-in of a point mutation (K46M) into exon 2 was introduced into HPK1 gene. **B**. Cytokine release after in vivo anti-CD3 treatment. HPK1 WT and KD mice were treated with 0.5mg/ml with anti-CD3. Serum was collected and analyzed for cytokine release 1.5hr post dosing. **C**. Proliferation of CD4+ T cells from spleen of HPK1 WT and KD mice. BrdU incorporation into dividing cells was used to measure T cell proliferation in vivo after immunization with OVA. OVA in CFA was administrated by subcutaneous injection. BrdU was administered in PBS by intraperitoneal injection. **D**. Proliferation of CD4+ T cells from lymph nodes of HPK1 WT and KD measured by BrdU incorporation.
(TIF)

S2 Fig OVA or KLH-induced in vivo antibody production. **A**. Levels of serum IgG_1_, IgG_2a_ and IgG_2b_ after initial and secondary challenge with OVA. **B**. Antibody production after in vivo challenge with KLH. Each mouse was immunized by i.p. injection with 250 μg of KLH dissolved in sterile saline. Blood for analysis was collected 14 days after the immunization to assess the anti-KLH IgM and IgG titers. N = 8 per group.
(TIF)

S3 Fig Effects of HPK1 KD on NK cells and DCs. **A**. Enhanced cytolytic activities of NK cells by HPK1 KD. NK cells were purified from spleen and cytolytic activities were evaluated by co-culture with NK sensitive YAC-1 cells as targets. **B**. Potentiation of CD8^+^ T cell proliferation by HPK1 KD bone marrow derived dendritic cells (BMDCs). DCs were generated with bone marrow cells from HPK1 WT and KD mice. The BMDCs were pulsed with OVA peptide and co-cultured with CFSE labeled naïve OVA specific CD8 ^+^ T cells from OVA specific TCR transgenic mice (OT1). The proliferation of CD8^+^ T cells were measured after 3 days of culture. All studies were repeated 3 times with representative data shown here.
(TIF)

S4 Fig Nanostring analysis of tumor draining lymph nodes from mouse sarcoma model. **A**. Genes up-regulated in tumor draining lymph nodes by HPK1 KD. **B**. Genes down-regulated in tumor draining lymph nodes by HPK1 KD. **C**. Pathway analysis. Pathway scores were fit using the first principal component of each gene set’s data. For simplicity, the scores for each sample (HPK1 KD or Vehicle, n = 5 per group) was averaged.
(TIF)

S5 Fig Body, organ weights, numbers of red blood cells and platelets in WT and HPK1 KD mice. (TIF)

S1 Table Organ weights from female and male of wild type and HPK1 KD mice. (TIF)
