# Supplementary figures and images for: Critical role of kinase activity of hematopoietic progenitor kinase 1 in anti-tumor immune surveillance
Source: PLoS One. 2019 Mar 26;14(3):e0212670. doi: 10.1371/journal.pone.0212670 (PMC6435129; doi:10.1371/journal.pone.0212670)

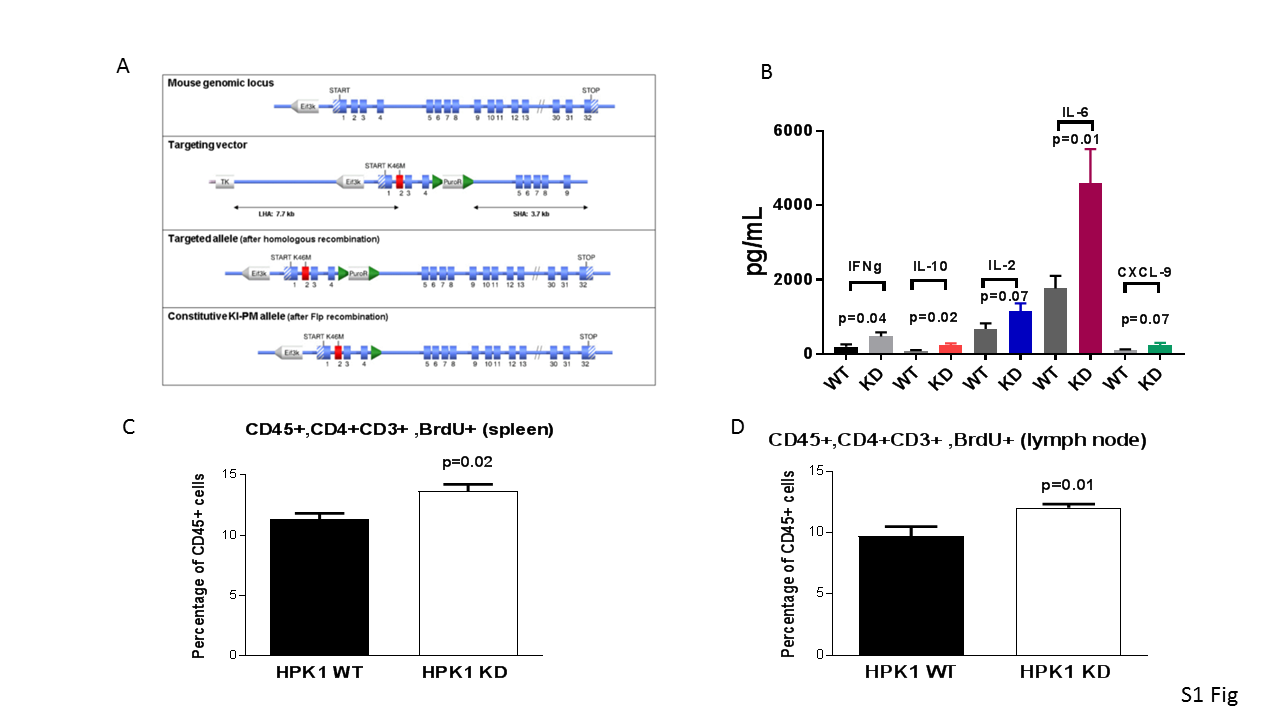

Supplement: S1 Fig — A. Scheme for the generation of HPK1 KD construct. A constitutive knock-in of a point mutation (K46M) into exon 2 was introduced into HPK1 gene. B. Cytokine release after in vivo anti-CD3 treatment. HPK1 WT and KD mice were treated with 0.5mg/ml with anti-CD3. Serum was collected and analyzed for cytokine release 1.5hr post dosing. C. Proliferation of CD4+ T cells from spleen of HPK1 WT and KD mice. BrdU incorporation into dividing cells was used to measure T cell proliferation in vivo after immunization with OVA. OVA in CFA was administrated by subcutaneous injection. BrdU was administered in PBS by intraperitoneal injection. D. Proliferation of CD4+ T cells from lymph nodes of HPK1 WT and KD measured by BrdU incorporation. (TIF) [file pone.0212670.s001.TIF]

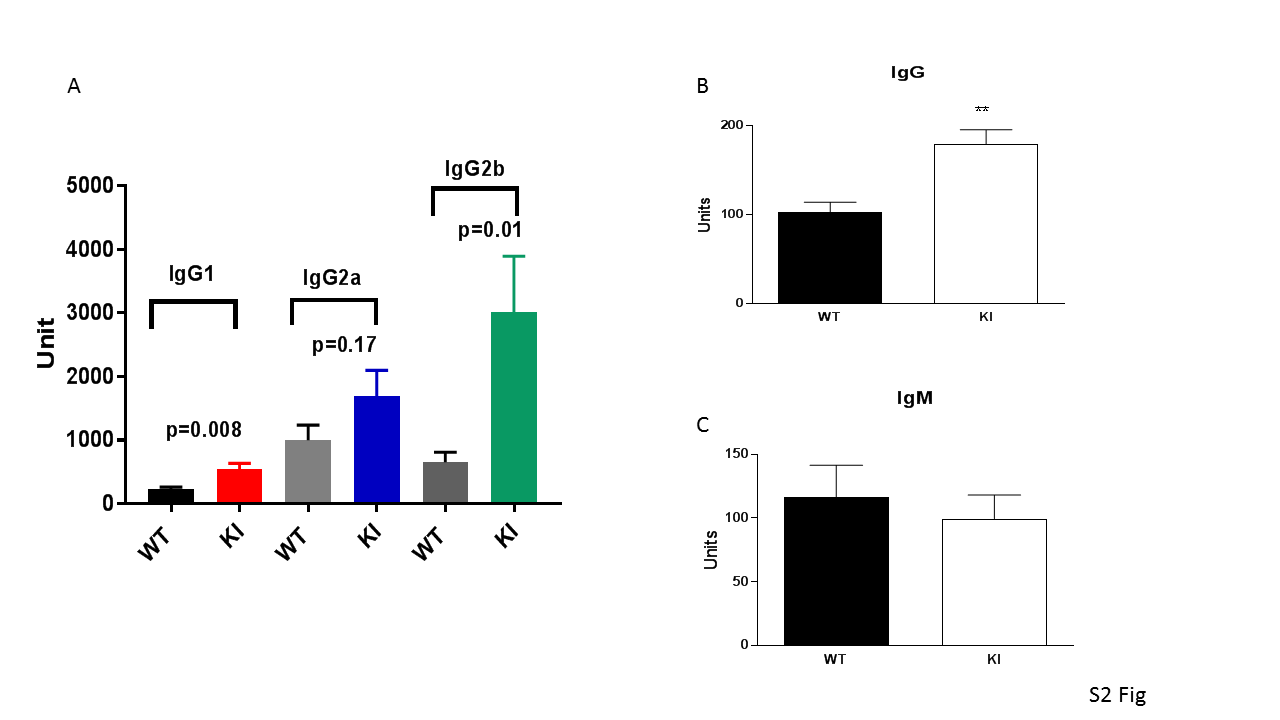

Supplement: S2 Fig — A. Levels of serum IgG1, IgG2a and IgG2b after initial and secondary challenge with OVA. B. Antibody production after in vivo challenge with KLH. Each mouse was immunized by i.p. injection with 250 μg of KLH dissolved in sterile saline. Blood for analysis was collected 14 days after the immunization to assess the anti-KLH IgM and IgG titers. N = 8 per group. (TIF) [file pone.0212670.s002.TIF]

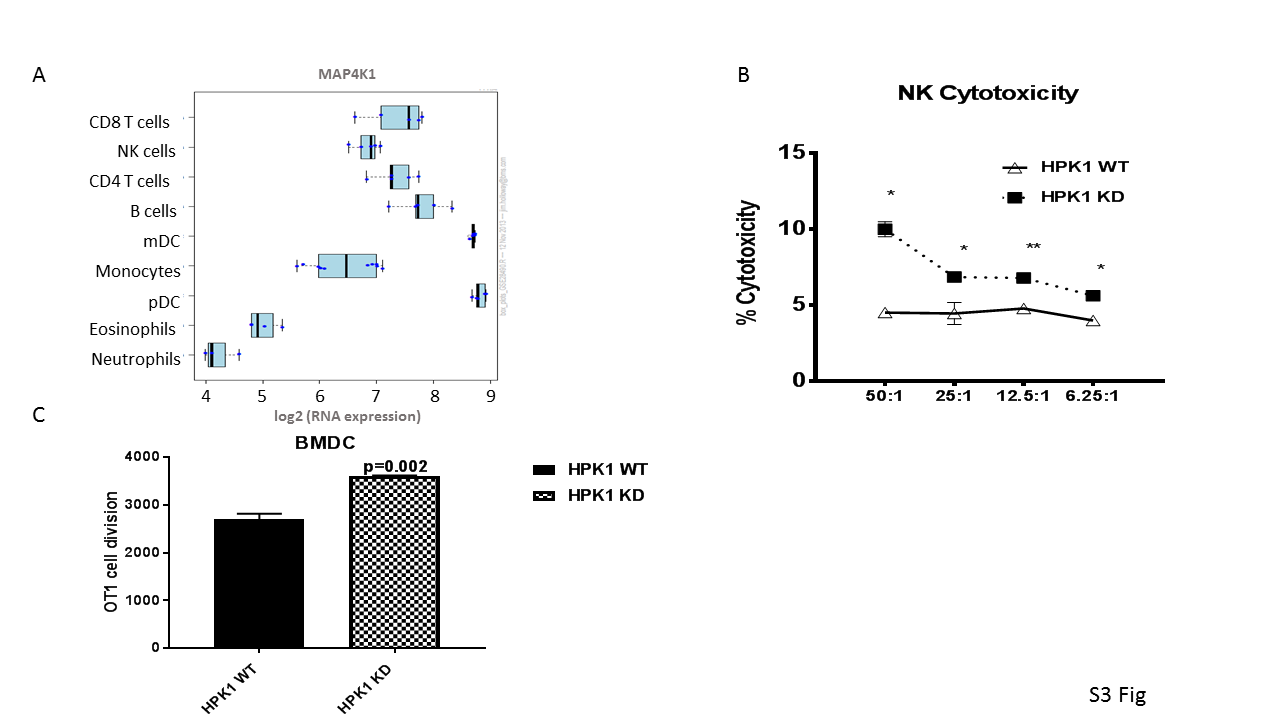

Supplement: S3 Fig — A. Enhanced cytolytic activities of NK cells by HPK1 KD. NK cells were purified from spleen and cytolytic activities were evaluated by co-culture with NK sensitive YAC-1 cells as targets. B. Potentiation of CD8+ T cell proliferation by HPK1 KD bone marrow derived dendritic cells (BMDCs). DCs were generated with bone marrow cells from HPK1 WT and KD mice. The BMDCs were pulsed with OVA peptide and co-cultured with CFSE labeled naïve OVA specific CD8 + T cells from OVA specific TCR transgenic mice (OT1). The proliferation of CD8+ T cells were measured after 3 days of culture. All studies were repeated 3 times with representative data shown here. (TIF) [file pone.0212670.s003.TIF]

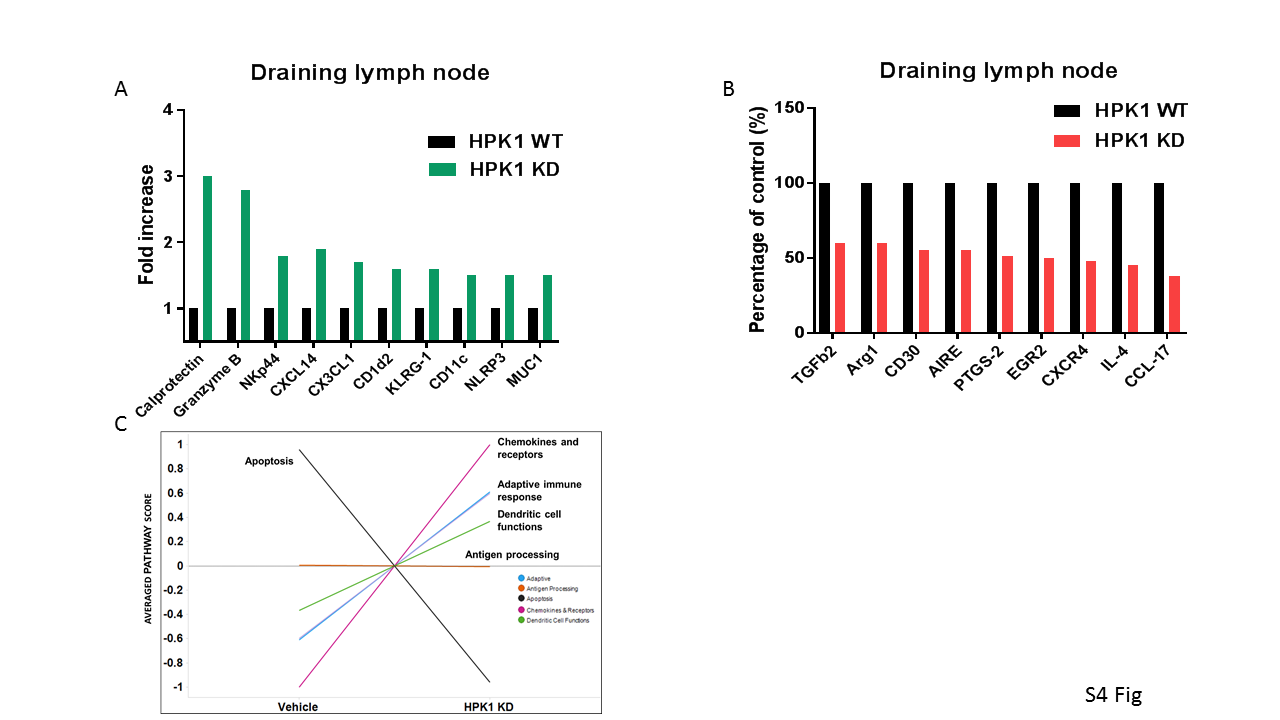

Supplement: S4 Fig — A. Genes up-regulated in tumor draining lymph nodes by HPK1 KD. B. Genes down-regulated in tumor draining lymph nodes by HPK1 KD. C. Pathway analysis. Pathway scores were fit using the first principal component of each gene set’s data. For simplicity, the scores for each sample (HPK1 KD or Vehicle, n = 5 per group) was averaged. (TIF) [file pone.0212670.s004.TIF]

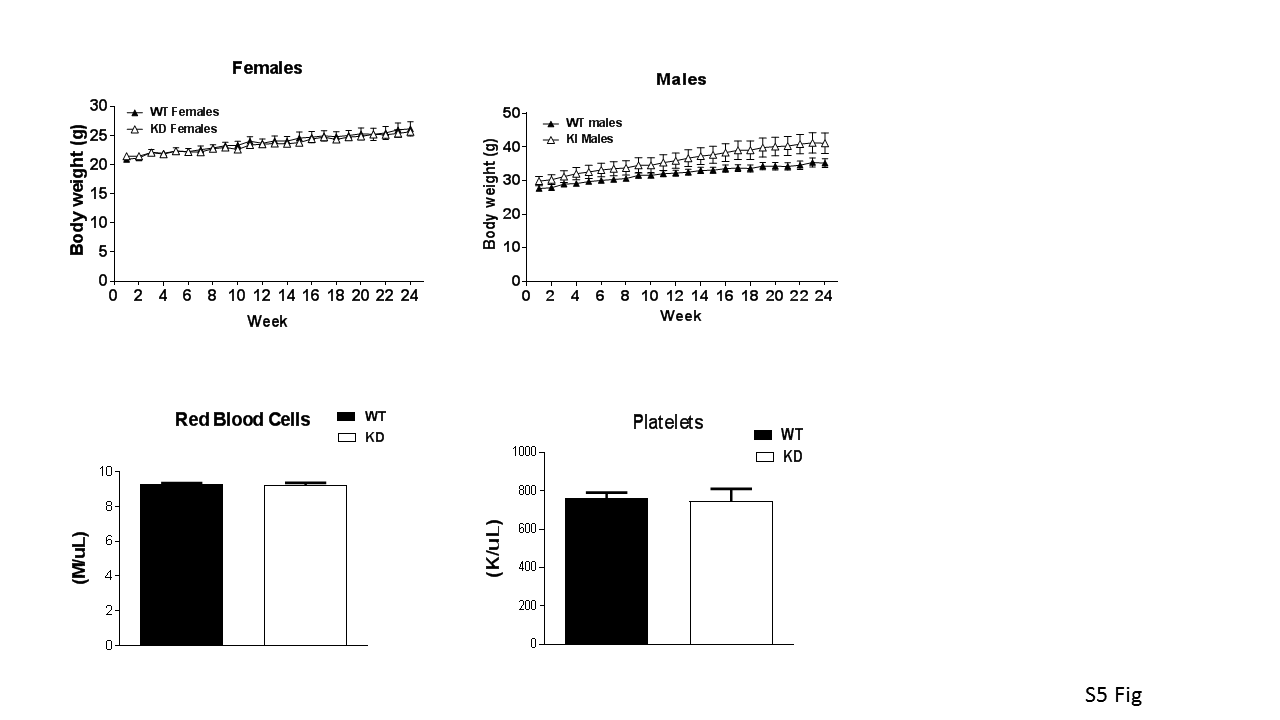

Supplement: S5 Fig — (TIF) [file pone.0212670.s005.TIF]

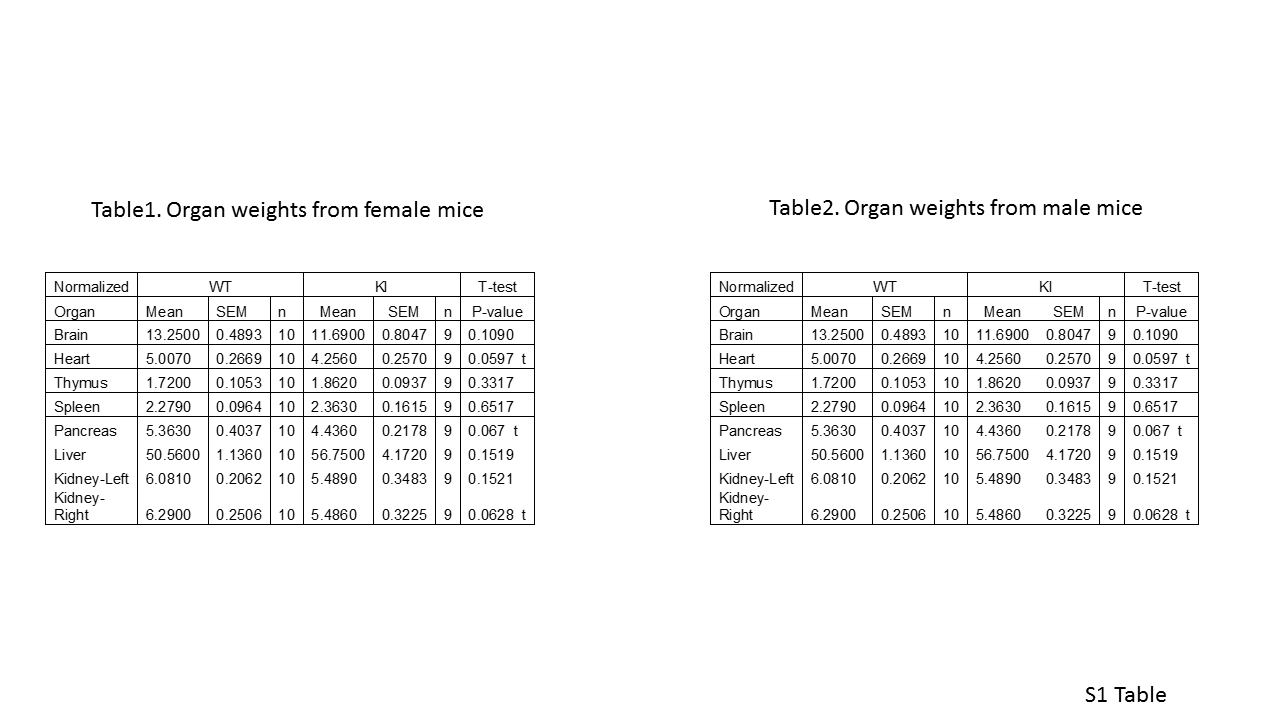

Supplement: S1 Table — (TIF) [file pone.0212670.s006.TIF]
